# Rapid Heating-Driven Variant Selection and Martensitic Refinement for Superior Strength–Ductility Synergy

**DOI:** 10.3390/ma18112488

**Published:** 2025-05-26

**Authors:** Siming Huang, Liejun Li, Haixiao Ye, Xianqiang Xing, Jianping Ouyang, Zhuoran Li, Xinkui Zhang, Songjun Chen, Zhengwu Peng

**Affiliations:** 1 National Engineering Research Center of Near-Net Shape Forming Technology for Metallic Materials, South China University of Technology, Guangzhou 510640, China; 2 Guangdong Orsa Steel Wire Co., Ltd., Guangzhou 511450, China; 3 School of intelligent Manufacturing, Guangzhou Vocational College of Technology & Business, Guangzhou 511442, China

**Keywords:** rapid heating, martensitic transformation, variant selection, prior austenite grain, grain boundary

## Abstract

This study elucidates the influence of rapid heating (300 °C/s) on martensitic transformation pathways, crystallographic variant selection, and the resulting mechanical performance in a medium-carbon steel. Compared with conventional heating, rapid heating markedly refines the prior austenite grain (PAG) and martensitic substructures, reducing the mean PAG size from 16.08 μm to 5.06 μm and the martensitic block size from 4.24 μm to 2.41 μm. The accelerated austenitizing and quenching promote a higher density of high-angle grain boundaries (HAGBs) and favor variant selection dominated by the closely packed (CP) group. Σ3 twin boundaries are also found to assist variant nucleation and contribute to microstructural complexity. Despite a marginal decrease in tensile strength, rapid-heated steels exhibit significantly enhanced ductility and a 28.3% increase in the product of strength and elongation (PSE) compared to their conventionally treated counterparts. These findings demonstrate that rapid heating not only enables effective refinement of martensitic substructures but also offers a powerful means of controlling variant evolution, thereby achieving a superior strength–ductility synergy in martensitic steels.

## 1. Introduction

Martensite, widely recognized for its exceptional strength and hardness, is a pivotal microstructural constituent in advanced high-strength steels. The mechanisms governing its formation have long been a central topic in physical metallurgy, offering essential insights for developing ultrahigh-strength steels with improved ductility and toughness [1,2,3,4,5,6]. Among various heat treatment strategies, quenching-based processes—particularly induction quenching—have emerged as efficient and scalable methods for promoting martensitic transformation by rapidly heating materials to austenitization temperatures followed by quenching [7,8,9,10,11]. Unlike conventional furnace heating, fast induction heating often achieves rates exceeding 300 °C/s, inducing non-equilibrium thermal conditions that significantly influence austenite evolution and subsequent martensitic transformation behavior [12].

In hypoeutectoid steels, austenitization typically proceeds in three sequential stages: dissolution of pearlite, expansion of austenite into ferrite, and homogenization of alloying elements [13,14]. However, under rapid heating, the extremely short thermal duration can result in incomplete austenitization, characterized by partially transformed microstructures, undissolved carbides, inhomogeneous element distribution, and retained ferrite [15,16,17]. Such non-equilibrium features alter the nucleation and growth of martensite, influencing not only its morphology but also its crystallographic variant distribution and mechanical performance [18].

Martensitic transformation is a diffusionless shear process, wherein face-centered cubic (FCC) austenite transforms into body-centered cubic (BCC) or body-centered tetragonal (BCT) martensite. This transformation obeys specific orientation relationships, most commonly the Kurdjumov–Sachs (K–S) orientation in steels with carbon content below 0.6 wt.%: {111}γ//{110}α and 110⟩γ//⟨111⟩α [19,20,21,22]. Under the K–S orientation, a single austenite grain can transform into 24 possible martensitic variants, as illustrated in Table 1 [23,24]. These variants can be grouped into three Bain groups based on crystallographic distortion or into four closely packed (CP) groups based on austenite habit planes [25,26]. Variants within the same Bain group share low misorientations (<15°), forming low-angle grain boundaries (LAGBs), whereas variants spanning different Bain groups tend to form high-angle grain boundaries (HAGBs), especially across CP groups where misorientations can exceed 50°. Consequently, variant selection directly influences the grain boundary character and, thereby, the mechanical properties of martensitic steels.

Grain refinement has been shown to enhance variant selectivity and transformation behavior. As reported by Wu [27] and Zhao et al. [28], decreasing the size of prior austenite grains (PAGs) narrows the pool of accessible variants, promoting selective transformation within specific CP groups and elevating the fraction of HAGBs. Furthermore, smaller PAGs and higher carbon content contribute to greater austenite stability and lower martensitic start temperatures, increasing the driving force for variant pair formation, particularly for self-accommodating pairs such as V1/V2, which reduce transformation strain [29,30,31]. These specific variant pairs exhibit superior lattice matching and interfacial stability, thereby enhancing mechanical performance.

Despite growing industrial adoption of rapid heating technologies, particularly in induction-hardening lines, the fundamental understanding of martensitic transformation under ultra-fast heating remains limited. Most studies to date have focused on conventional heating conditions, while the effects of rapid austenitization on variant selection mechanisms, microstructural refinement, and crystallographic boundary formation are still largely unexplored—especially at heating rates above 300 °C/s. Key questions persist regarding how rapid heating alters variant population dynamics, misorientation distributions, and their correlations with strength–ductility synergy.

In this study, we systematically investigate the effects of rapid heating on martensitic transformation behavior, variant selection, and mechanical performance in a medium-carbon steel. By employing electron backscatter diffraction (EBSD), scanning electron microscopy (SEM), ORTools, and tensile testing, we examine the evolution of grain structure, crystallographic misorientation, variant grouping, and the resulting strength–ductility relationship. Our findings reveal that rapid heating not only induces significant refinement of PAGs and martensitic substructures but also promotes CP group-dominated variant selection and increases the density of HAGBs. These microstructural changes lead to a substantial improvement in the product of strength and elongation (PSE), offering new insights into the transformation pathways and performance optimization of martensitic steels under non-equilibrium thermal conditions.

## 2. Materials and Methods

### 2.1. Materials and Heat Treatment Process

**Figure 1:**
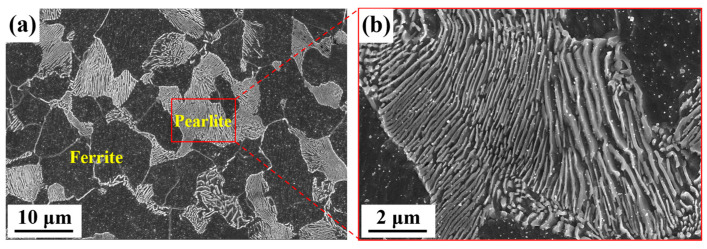
SEM micrographs of original microstructure of the experimental material: (**a**) cold-drawn material, (**b**) lamellar pearlite.

The material used in this study is a typical medium-carbon steel, provided by Guangxi Liuzhou Iron and Steel Group Co., LTD (Liuzhou, Guangxi, China), with its chemical composition listed in Table 2. The steel was supplied as hot-rolled wire rods with an initial diameter of 12 mm, which were then cold-drawn to a final diameter of 10.7 mm. The initial microstructure comprised approximately 40% pearlite and 60% ferrite, as illustrated in Figure 1.

**Figure 2:**
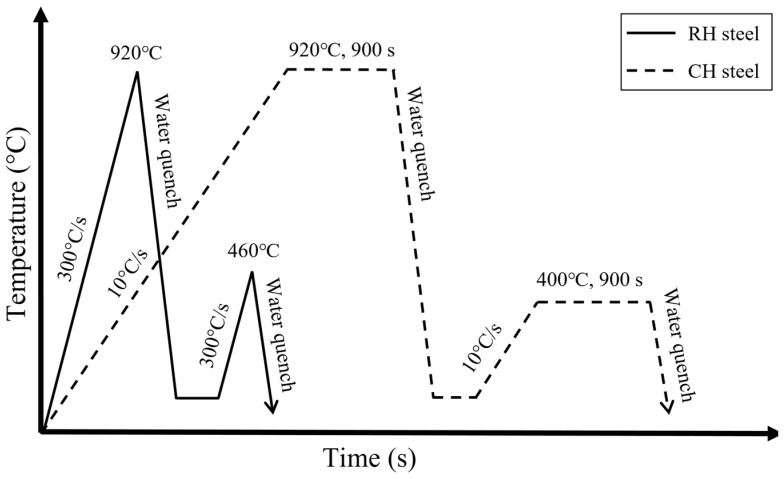
Heat treatment process flow diagram of studied steels.

The heat treatment of the experimental steel was conducted using an advanced rapid induction heating production line provided by Guangdong Yima Holdings Group Co., Ltd. (Zhongshan, China), with a heating rate of about 300 °C/s. Water spray was used for cooling during both quenching and tempering. The steel was austenitized at ~920 °C, immediately followed by quenching, and then tempered at ~460 °C without holding time. Samples processed under these conditions were designated as RH steel. To compare the microstructures under different heating methods, a conventional heating experiment was performed using a tubular resistance furnace (GSL-1500X-OTF, Kejing, Hefei, Anhui, China). The heating rate in this process was ~10 °C/s, with water cooling employed for both quenching and tempering. Austenitization and tempering temperatures were set to 920 °C and 400 °C, respectively, with a holding time of 900 s. Samples treated under these conditions were designated as CH steel. The heat treatment procedures for the steels are shown in Figure 2.

### 2.2. Microstructural Characterization

To examine the microstructure, metallographic samples were ground, polished, and etched using a 4 vol.% nital solution. The microstructure was observed by scanning electronic microscopy (SEM, Sigma 300, ZEISS, Oberkochen, Baden-Württemberg, Germany), while Image-J software (version 1.54 g) quantified the ferrite and martensite volume fractions. Tensile tests were performed at room temperature on a UTM5305 universal testing machine (SUNS, Shenzhen, China) equipped with an extensometer (Model 3442, Epsilon, Jackson, WY, USA), with a strain rate of 10^−3^ s^−1^. The tensile specimens, with a gauge length of 15 mm and a diameter of 3 mm, were prepared from the tempered specimens and aligned parallel to the cold-drawn direction. At least five independent tensile tests were conducted for each material to ensure the reproducibility of the mechanical properties.

Crystallographic characterization was conducted using an Nova NanoSEM430 (FEI, Hillsboro, OR, USA) field emission scanning electron microscope equipped with an Symmetry S2 EBSD detector (Oxford Instruments, Oxfordshire, Abingdon, UK). Data was collected at an acceleration voltage of 20 kV, with a step size of 0.06 μm. After mechanical polishing, samples were electrochemically polished at 20 V using a solution of 90 vol.% acetic acid and 10 vol.% perchloric acid to eliminate surface stress. AztecCrystal (version 2.1), MTEX (version 5.10.2), and ORTools (version 2.3.0) were utilized for misorientation analysis, parent austenite grain reconstruction, and other data processing tasks. ORTools, an add-on for the crystallographic analysis tool MTEX, was employed for orientation relationship (OR) discovery, advanced OR and variant analysis, as well as parent phase grain reconstruction [32].

## 3. Results and Discussion

### 3.1. Microstructural Evolution Under Rapid Heating

**Figure 3:**
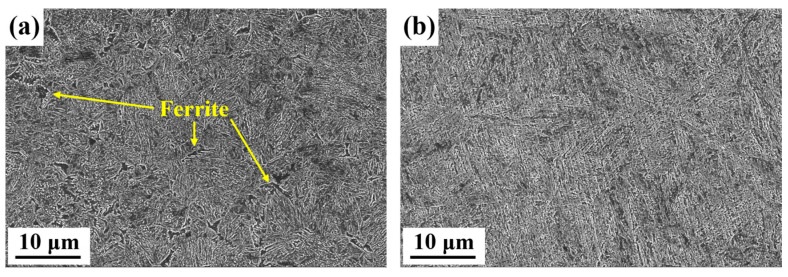
SEM micrographs of the samples after heat treatment: (**a**) RH steel, (**b**) CH steel.

Rapid induction heating, characterized by a high heating rate and short holding time, may cause incomplete austenitization, non-uniform microstructure, and uneven elemental distribution in quenched samples. These factors significantly affect martensitic transformation, leading to noticeable differences in microstructure and crystallographic morphology compared to tubular furnace-treated samples. Figure 3 illustrates the microstructure of the samples. The matrix of RH steel is martensite, with a small amount of ferrite also observed, as indicated by the yellow arrows in Figure 3a and quantified to be approximately 8.6% using Image-J software. In contrast, the microstructure of CH steel is nearly fully martensitic.

**Figure 4:**
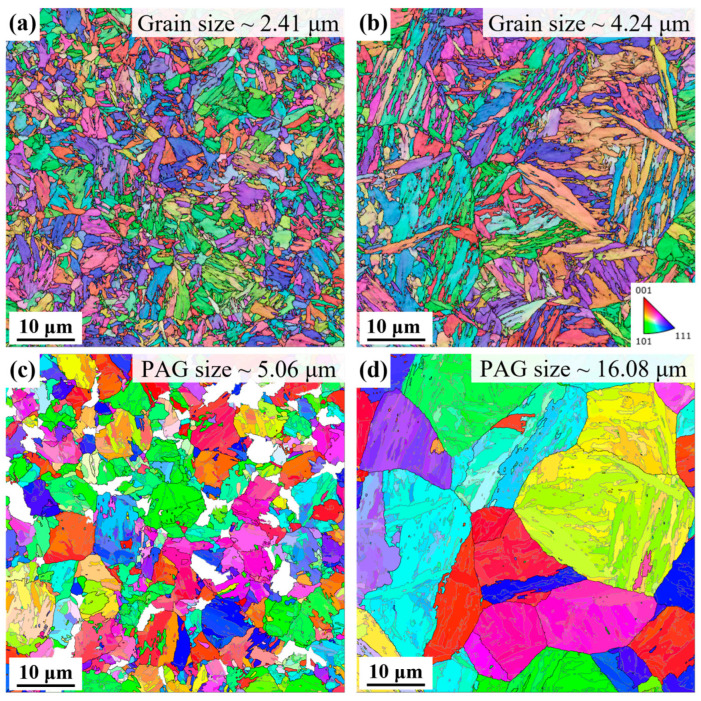
(**a**,**b**) EBSD inverse pole figure with band contrast of the samples; (**c**,**d**) prior austenite maps of the samples after parent phase reconstruction (blank areas represent residual ferrite): (**a**,**c**) RH steel, (**b**,**d**) CH steel.

Figure 4 presents inverse pole figures (IPFs), revealing the crystallographic orientation relationships of grains. Each pixel in the IPF maps represents Euler angle information aligned with spatial coordinates. In Figure 4a,b, the traces of martensitic block boundaries correspond to the intersections of {111} planes of parent austenite with the observation surface. Grain boundaries in RH steel are more irregular than the relatively straight boundaries in CH steel, likely due to differences in PAG size and nucleation energy. RH steel exhibits small, fragmented blocks, whereas CH steel shows elongated, bamboo-leaf-like structures with occasional coarse blocks. The area-weighted equivalent circular diameters of grains in RH and CH steel are 2.41 μm and 4.24 μm, respectively.

Ferrite and martensite were identified and classified using EBSD data based on parameters such as band contrast, band slope, and mean angular deviation (MAD) values. The prior austenite was then reconstructed as the parent phase. Figure 4c,d shows the area-weighted equivalent circular diameters of PAGs in RH and CH steel as 5.06 μm and 16.08 μm, respectively, with significantly smaller PAGs in RH steel.

The studied hypoeutectoid steel, initially composed of a pearlite-ferrite mixture, begins austenitization in the pearlite regions. With increasing temperature, carbon atoms diffuse into ferrite at the ferrite–austenite interfaces, facilitating the transformation of ferrite into austenite. Austenitization progresses through interface expansion, accompanied by boundary migration and grain growth within the austenite. In the early stages, the nucleation rate of austenite exceeds the boundary migration rate, increasing austenite nuclei that eventually merge and homogenize during transformation [33].

In RH steel, the rapid heating rate creates greater superheat and numerous nucleation sites. High-energy boundaries enable complete austenitization in manganese- and carbon-rich pearlite regions. However, ferrite–austenite boundaries leave some ferrite regions untransformed, resulting in residual ferrite. Residual ferrite segments large austenite grains, restricting their growth. Additionally, the short heating duration preserves the early growth state of austenite, leading to incomplete grain boundary migration and smaller, irregularly shaped PAGs with non-uniform microstructures. In CH steel, the slower heating rate approximates equilibrium transformation, providing ample time for austenite grains to grow and homogenize. Complete austenitization occurs, with austenite grain boundaries merging during growth, forming more uniform and flat boundaries. Quenching with water spray induces martensitic transformation in the fully austenitized regions of both RH and CH steel, generally adhering to the K–S relationship.

**Figure 5:**
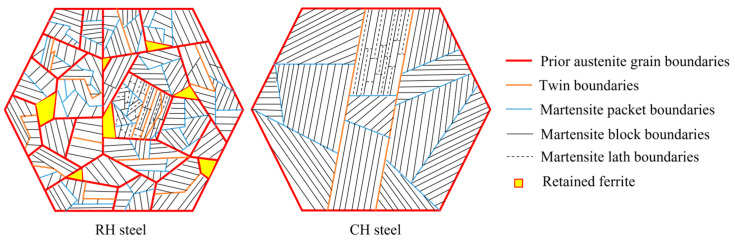
Schematic microstructure of RH steel and CH steel (red lines represent PAGBs, orange lines represent twin boundaries, blue lines represent packet boundaries, black lines represent block boundaries, black dashed lines represent lath boundaries, yellow areas represent residual ferrite).

Figure 5 depicts the microstructural characteristics of the samples. PAG diameters in RH steel range from 3 to 10 μm, while those in CH steel range from 10 to 20 μm. Key differences are as follows: (1) RH steel has smaller PAGs and martensitic packet areas than CH steel; (2) PAGBs and twin boundaries in RH steel appear more irregular; (3) RH steel contains residual intergranular ferrite and fewer packets per PAG compared to CH steel; (4) the aspect ratio of martensitic blocks is smaller in RH steel.

### 3.2. Analysis of Misorientation and Martensitic Variant Pairs

**Figure 6:**
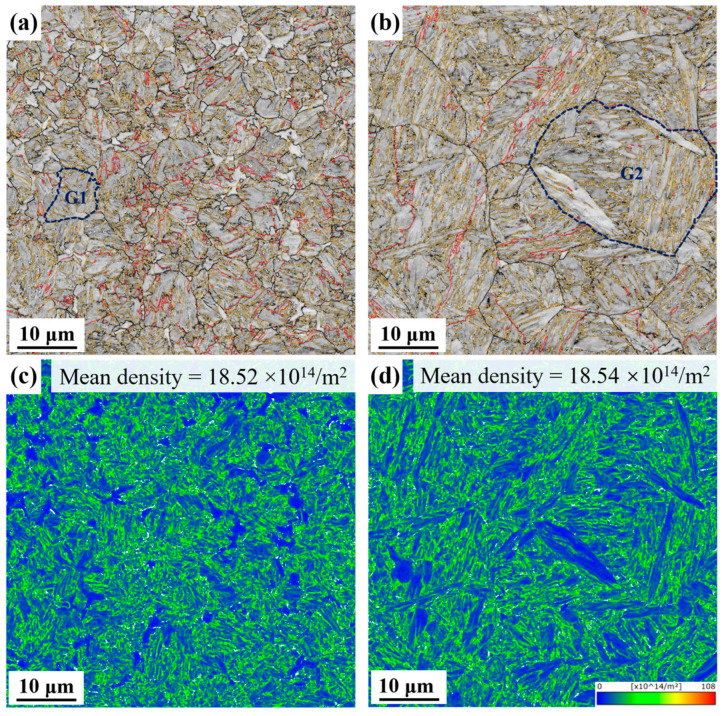
GB maps of the samples (white lines: 2° < θ < 15°, black lines: 15° < θ < 45°, yellow lines: 45° < θ, red lines: Σ3 grain boundaries), G1 and G2 represent two typical PAGs; GND maps of the samples: (**a**,**c**) RH steel, (**b**,**d**) CH steel.

The distribution of misorientation, including grain boundaries and variant pairs, is closely linked to the microstructure and directly influences the material’s mechanical properties. Figure 6a,b shows the EBSD grain boundary (GB) maps for samples subjected to different heat treatment processes. Misorientation angles are classified as low angle grain boundaries (LAGBs, 2–15°) and high angle grain boundaries (HAGBs, 15–60°), with 15–45° specifically attributed to prior austenite grain boundaries (PAGBs) [34]. Two representative PAGs (G1, G2) are marked. Figure 6a,b also highlights the twin boundaries, identified as Σ3 boundaries in the coincident site lattice (CSL) theory, a subset of special HAGBs [35]. In RH steel, twin boundaries display irregular distribution. In contrast, CH steel exhibits more typical, straight twin boundaries that extend through the PAGs.

Table 3 provides statistical data on the fraction and density of boundary types. Boundary density, defined as the boundary length divided by the grain area, reflects the actual boundary distribution in the samples. The LAGB fractions in RH and CH steel are nearly identical. However, RH steel shows significantly higher PAGB fractions and densities compared to CH steel. This is attributed to the refined PAGs in RH steel, which increase the number of PAGs. The overall boundary density is higher in RH steel, reflecting its finer grains, greater number of grain boundaries, and more tortuous and irregular boundary morphologies, which together constitute a more complex microstructure. The higher boundary density in RH steel may impede dislocation motion, enhancing both strength and ductility.

Geometrically necessary dislocations (GNDs) in both samples were analyzed, as shown in Figure 6c,d. The mean GND density is 18.52 × 10^14^/m^2^ in RH steel and 18.54 × 10^14^/m^2^ in CH steel. Although the heating methods differ, the dislocation density in both samples are nearly identical after water quenching.

**Figure 7:**
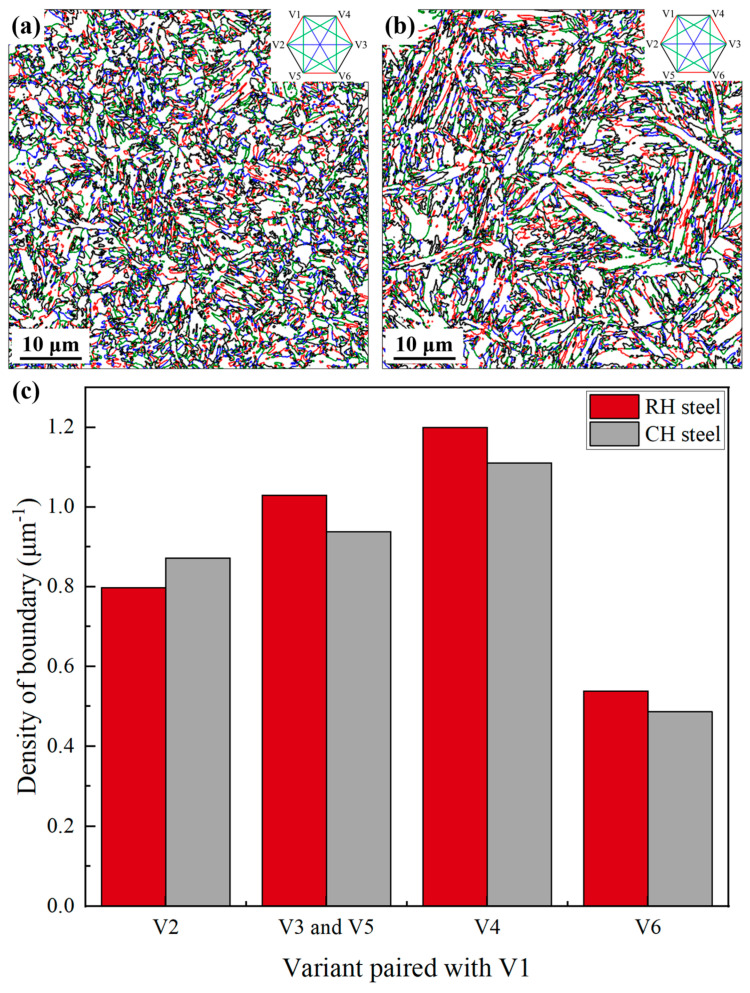
Statistical analysis of martensite variant pairs; (**a**,**b**) distribution of variant pairs in RH steel and CH steel; (**c**) density statistics of variant pairs.

In lath martensite, the CP group corresponds to a packet, with each variant representing a martensite block [36]. Since a packet contains many blocks, block boundaries have a greater influence than packet boundaries [37]. Therefore, this study calculated the equivalent variant pair distribution by transforming and aligning the crystal orientations of CP 2, CP 3, and CP 4 with CP 1 [34,38]. Variant pairs were identified and analyzed using ORTool, as shown in Figure 7. Martensite boundaries were extracted and classified into four types based on axis/angle relationships: V1/V2, V1/V3 (and V5), V1/V4, and V1/V6. Density analysis showed that RH steel had a slightly lower density of V1/V2 variant pairs compared to CH steel, but higher densities of V1/V3 (and V5), V1/V4, and V1/V6 variant pairs.

Refined PAGs generally enhance stability and increase transformation strain during martensitic transformation. Variant pairs like V1/V2, which accommodate transformation strain, are expected to form preferentially [29,30]. However, the results of this study slightly deviate from this trend, likely due to the effects of alloying elements, phase transformation space, and residual ferrite, which constrain martensitic transformation pathways, nucleation, and growth [31]. On the one hand, RH steel, characterized by smaller PAGs, has shorter martensitic shear distances. In CH steel, larger PAGs increase shear distances, accumulating more transformation strain and promoting the formation of V1/V2 variant pairs to release the strain. On the other hand, residual ferrite along the PAGBs in RH steel, as a softer phase, may release transformation strain during martensitic transformation, exerting a more significant effect than V1/V2 variant pairs. This explains why RH steel exhibits fewer V1/V2 variant pairs than CH steel.

Variant pairs were further analyzed based on their misorientation characteristics. The V1/V4 variant pair, with a misorientation angle of 10.5°, corresponds to sub-lath block boundaries and belongs to LAGBs, whereas the other variant pairs belong to HAGBs. Notably, the V1/V2 variant pair corresponds to twin boundaries, and the V1/V3 and V1/V5 are equivalent pairs with a 60° rotation around the <110> axis [39]. The statistical results are consistent with the EBSD boundary analysis, confirming that RH steel exhibits higher boundary density than CH steel, especially in HAGBs. This indicates that RH steel may possess superior overall mechanical properties compared to CH steel.

### 3.3. Martensitic Variants Selection

Martensitic transformation is a diffusionless phase transition. According to the phenomenological theory of martensite crystallography (PTMC), this transformation involves adaptive shear deformation of austenite to form martensite in the most favorable manner [40]. Factors such as PAG morphology, undercooling degree, carbon content, and stress state influence variant selection during martensitic transformation [41,42,43]. Among these factors, PAG morphology plays a crucial role in determining martensite variant selectivity. Differences in PAG morphology result in significantly varied variant distributions and traces between RH steel and CH steel.

**Figure 8:**
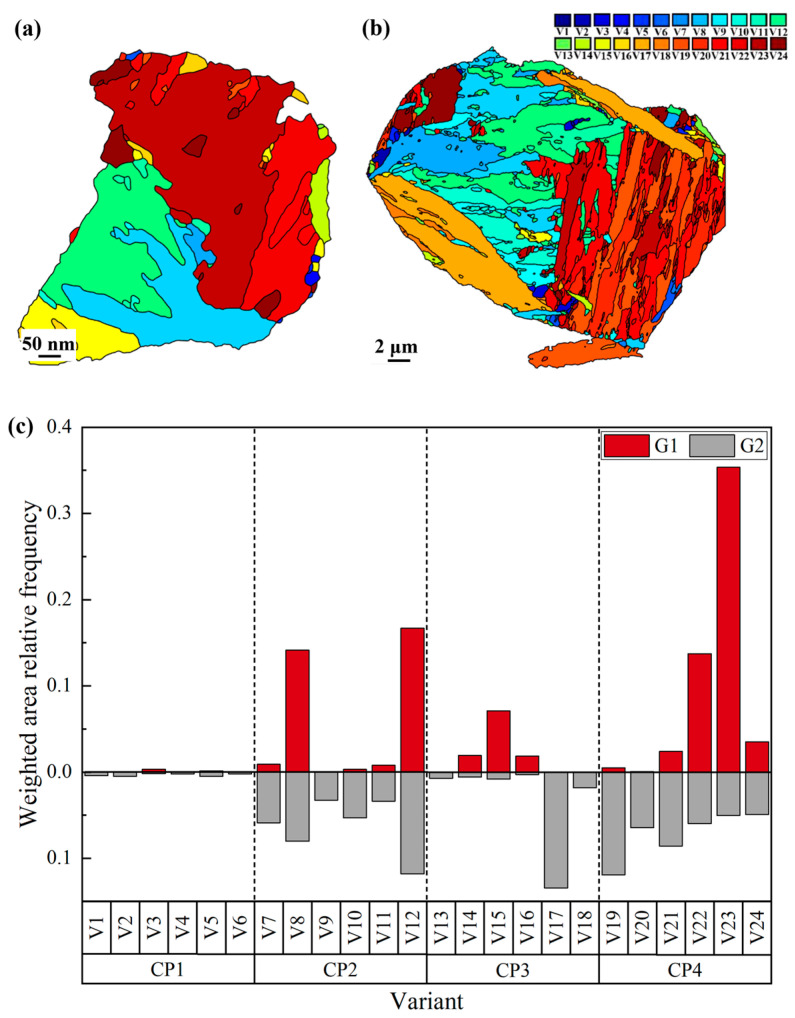
Martensite variant statistics of G1 and G2; (**a**,**b**) distribution map of martensite variants in G1 and G2; (**c**) the frequency of 24 variants in G1 and G2.

In this study, two representative PAGs, G1 and G2 (Figure 6a,b), were selected for statistical analysis of variant distributions, with the results shown in Figure 8. In G1, the variants are primarily block-shaped, with specific variants like V8, V12, V22, and V23 exhibiting marked dominance. Other variants appear in smaller proportions, with V1, V2, V17, and V18 completely absent. In contrast, the variants in G2 are mostly lamellar-shaped, exhibit a more uniform distribution, and display greater variant diversity. These results indicate that the distribution of variants in RH steel is highly selective, with certain variants preferentially forming.

Variant selection in martensitic transformation is primarily influenced by the CP group and Bain group. A key characteristic of CP group-dominated variant selection is the significantly higher proportion of a single CP group compared to others, resulting in better continuity of martensite packets. Similarly, Bain group-dominated variant selection is marked by the dominance of one Bain group over others.

**Figure 9:**
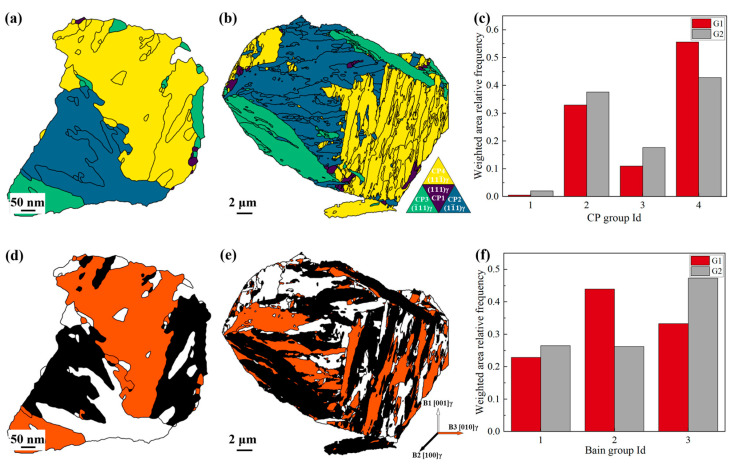
The statistics of dominant variant groups; (**a**,**b**) distribution map of the CP group; (**d**,**e**) distribution map of the Bain group: (**a**,**d**) G1, (**b**,**e**) G2. (**c**,**f**) The frequency of CP group and Bain group in G1 and G2.

The statistical analysis of G1 and G2 based on CP group and Bain group is shown in Figure 9. Figure 9a–c illustrates the CP group distribution, indicating that both PAGs primarily consist of three CP groups. Among these, CP 2 and CP 4 exhibit higher proportions, while CP 1 is nearly absent, suggesting CP 1 is less likely to form and CP 2 and CP 4 are more favorable. This implies that variant selection in both samples is governed by CP groups. Notably, CP 4 in G1 shows a significantly higher proportion compared to other CP groups in G1 and G2, indicating a more pronounced CP group dominance in RH steel relative to CH steel. Figure 9d–f illustrates Bain group distributions, showing similar proportions of all Bain groups in both samples. No clear evidence of Bain group-dominated variant selection is observed. These findings align with previous studies. As noted earlier, the finer and more stable PAGs in RH steel result in a more pronounced CP group-dominated variant selection.

Additionally, during the high-temperature austenitization stage, austenitic twin may form. Twin boundaries (Σ3 boundaries) significantly influence variant selection due to their low energy and unique geometric symmetry [44]. Figure 6a,b shows trace lines of martensitic packets parallel to Σ3 grain boundaries, indicating that straight twin boundaries induce packet alignment parallel to them.

**Figure 10:**
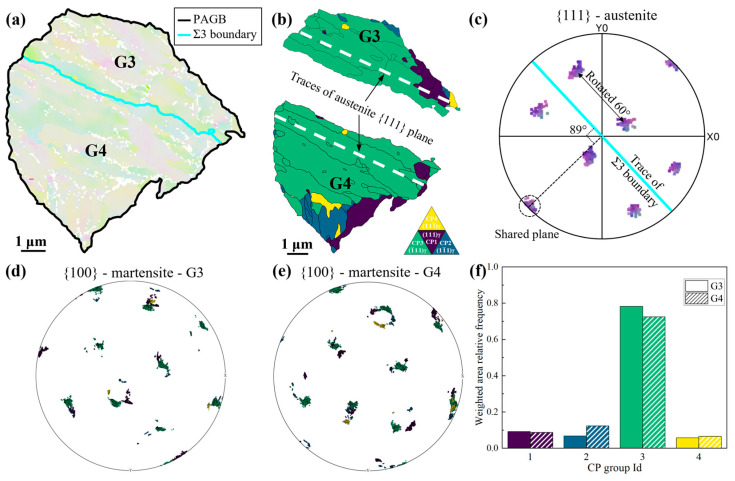
Inducing effect of Σ3 grain boundaries on variant selection; (**a**) GB map of a PAG region; (**b**) CP group distribution map of G3 and G4; (**c**) pole figure of the {111} plane of austenite; (**d**,**e**) pole figure of the {100} plane of martensite in G3, G4; (**f**) the frequency of the CP groups in G3 and G4.

A PAG region in RH steel was selected for analysis. Figure 10a displays the reconstructed PAG, with the austenite divided into G3 and G4 regions by the Σ3 grain boundary. The boundary between G3 and G4 exhibits an <111>/60° axis/angle relationship based on crystallographic orientation, confirming it as a Σ3 twin boundary. Figure 10b classifies the packets in G3 and G4, with trace lines (white dashed lines) of the austenite {111} planes drawn for each region. The packet traces near the Σ3 boundary, the trace lines of the austenite {111} planes, and the Σ3 boundary itself are nearly parallel.

Figure 10c shows the {111} pole figure of austenite, where overlapping poles between G3 and G4 indicate a shared {111} plane. Pole figure measurements reveal an 89° angle between the line connecting the shared plane to the pole figure center and the Σ3 boundary trace line, confirming that the shared plane is nearly parallel to the Σ3 boundary. Figure 10d,e depicts the {100} pole figures of martensitic variants in G3 and G4, where pole colors denote different packets. Overlapping green regions in G3 and G4 further confirm the shared plane between the two regions. Figure 10f shows CP group distribution statistics for G3 and G4, highlighting CP 3 as the dominant group in both regions. As shown in Figure 10b, CP 3 corresponds to packets nearest the Σ3 boundary, suggesting that the Σ3 boundary influences martensite selection.

In the studied steels, twin boundaries are typically straight and parallel to the {111} planes of adjacent austenite grains. During martensitic transformation, martensite preferentially nucleates on twin boundaries and grows by shear along both sides, requiring minimal energy for the process. Consequently, Σ3 twin boundaries strongly promote the formation of packets aligned parallel to them, influencing variant selection. Statistical analysis reveals that the Σ3 twin boundary lengths for RH steel and CH steel are 968 μm and 551 μm, respectively. This suggests that under rapid heating conditions, the influence of Σ3 twin boundaries on martensitic transformation is more pronounced.

In addition to the crystallographic character, the geometric morphology of Σ3 twin boundaries also influences variant formation. In RH steel, rapid austenitization leads to significantly smaller prior austenite grains and more irregular, tortuous Σ3 boundaries compared to the relatively straight twin boundaries observed in CH steel. These curved twin boundaries disrupt the growth continuity of martensitic packets and tend to promote the nucleation of blocky-shaped martensitic variants. As a result, RH steel exhibits a higher fraction of small, block-like martensitic structures. This effect, when considered alongside the finer PAGs and increased Σ3 boundary density in RH steel, further enhances CP group-dominated variant selection and contributes to the observed microstructural refinement.

### 3.4. Relationship Between Microstructure and Mechanical Properties

**Figure 11:**
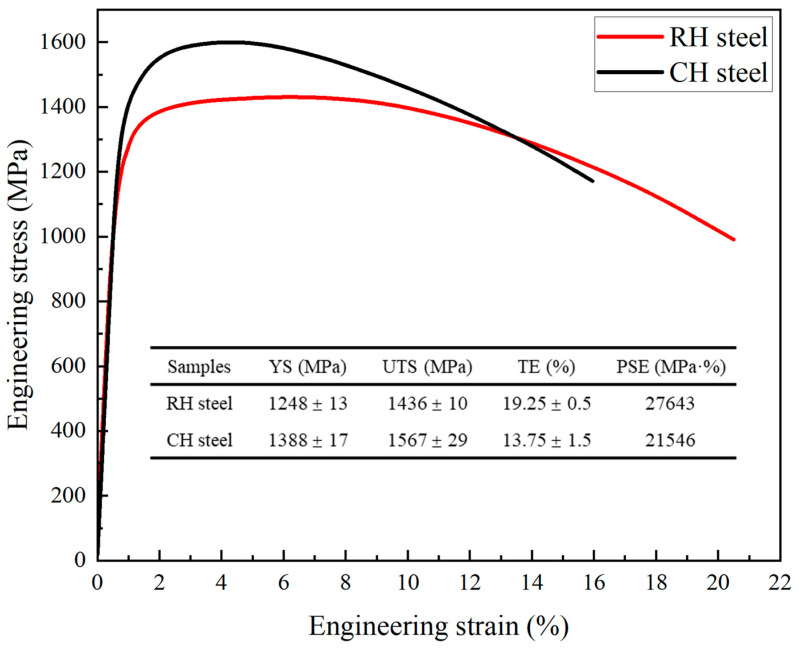
Tensile mechanical properties of the studied steels. YS, yield strength; UTS, ultimate tensile strength; TE, total elongation; PSE, the product of strength and elongation.

Figure 11 presents the engineering stress–strain curves and mechanical properties of the studied steels. The ultimate tensile strengths (UTSs) of RH steel and CH steel are 1436 MPa and 1567 MPa, respectively. RH steel exhibits a significantly higher total elongation (TE) of 19.25%, compared to 13.75% for CH steel. The product of UTS and TE is 27,643 MPa·% for RH steel and 21,546 MPa·% for CH steel, representing a 28.3% improvement for RH steel. This indicates that RH steel demonstrates superior overall mechanical performance [28,45].

Differences in mechanical properties arise from the microstructure and crystallographic features. RH steel contains a small amount of ferrite, which slightly reduces its strength. However, the dispersed ferrite acts as a soft phase, enabling substantial deformation and uniform stress distribution, thereby enhancing plasticity. In contrast, martensite, as a hard phase, resists large-scale deformation during tensile testing. Consequently, the presence of ferrite in RH steel significantly improves its plasticity compared to CH steel, especially in terms of uniform elongation.

Additionally, RH steel demonstrates stronger variant selectivity, with more pronounced CP group-dominated selection. As CP groups include variants from different Bain groups, RH steel exhibits a higher density of HAGBs [30]. HAGBs play a crucial role in impeding crack propagation. First, the large misorientation between adjacent grains causes cracks to deviate when crossing grain boundaries, consuming additional energy and slowing propagation. Second, HAGBs limit dislocation slip and form local plastic zones near crack tips, which absorb energy, blunt crack tips, and inhibit further propagation [31].

Among HAGBs, Σ3 grain boundaries (twin boundaries) play a unique role in enhancing steel’s plasticity matching. These boundaries not only redirect or terminate crack propagation, improving fracture toughness, but also, due to their low energy properties, facilitate better strain coordination between grains during deformation. This reduces dislocation pile-up and stress concentration compared to ordinary HAGBs, making Σ3 boundaries less prone to act as crack nucleation sites [45]. Consequently, RH steel, characterized by a higher density of HAGBs, demonstrates superior overall mechanical properties.

In conclusion, the superior mechanical properties of RH steel are primarily attributed to its favorable microstructure, especially its higher density of HAGBs, which enhance both plasticity and toughness. The presence of Σ3 twin boundaries further strengthens its crack resistance, significantly contributing to its superior performance compared to CH steel.

## 4. Conclusions

This study systematically investigated the effects of rapid versus conventional heating on martensitic transformation behavior, variant selection, and mechanical performance in a medium-carbon steel. The key findings are summarized as follows:

(1) Rapid heating at 300 °C/s significantly refined the microstructure, reducing the prior austenite grain (PAG) size from 16.08 μm to 5.06 μm and the martensitic block size from 4.24 μm to 2.41 μm, while retaining approximately 8.6% ferrite. This microstructural refinement reflects the non-equilibrium nature of rapid austenitization, which constrains transformation time and alters the austenite evolution process.
(2) The rapid austenitizing and quenching altered the crystallographic and morphological features of the martensitic structure, leading to a higher density of high-angle grain boundaries (HAGBs), irregular grain boundary morphology, and reduced variant diversity. Notably, rapid heating promoted variant selection dominated by closely packed (CP) groups and facilitated variant formation through Σ3 twin boundaries, enhancing microstructural complexity.
(3) These structural and crystallographic modifications translated into superior mechanical performance, with the rapid-heated specimens achieving a 28.3% higher product of strength and elongation (PSE) compared to conventionally heated counterparts. This improvement is attributed to the synergistic effects of refined martensitic substructures, increased HAGB density, and retained ferrite.

In summary, this work demonstrates that rapid heating is an effective strategy for tailoring martensitic transformation pathways and variant architecture, thereby enabling superior strength–ductility synergy. These insights contribute to a deeper mechanistic understanding of transformation behavior under extreme thermal conditions and offer guidance for the design of advanced heat treatment routes in high-performance steels. Future studies may extend this approach to other alloy systems and incorporate in-situ or post-mortem micro-crack path analysis, enabling a more direct evaluation of how martensitic variant selection influences fracture resistance and toughness.

## Figures and Tables

**Table 1:** The 24 martensitic variants in the K–S orientation relationship. Reprinted with permission from Ref. [24]. Copyright 2022 Elsevier.

Variant	Plane Parallel	Direction Parallel	CP Group	Bain Group	Misorientation From V1 (°)
V1	(111) γ//(011) α	[−101] γ//[−1−11] α	CP1	B1	-
V2		[−101] γ//[−11−1] α		B2	60.0
V3		[01−1] γ//[−1−11] α		B3	60.0
V4		[01−1] γ//[−11−1] α		B1	10.5
V5		[1−10] γ//[−1−11] α		B2	60.0
V6		[1−10] γ//[−11−1] α		B3	49.5
V7	(1−11) γ//(011) α	[10−1] γ//[−1−11] α	CP2	B2	49.5
V8		[10−1] γ//[−11−1] α		B1	10.5
V9		[−1−10] γ//[−1−11] α		B3	50.5
V10		[−1−10]γ//[−11−1] α		B2	50.5
V11		[011] γ//[−1−11] α		B1	14.9
V12		[011]γ//[−11−1] α		B3	57.2
V13	(−111) γ//(011) α	[0−11] γ//[−1−11] α	CP3	B1	14.9
V14		[0−11] γ//[−11−1] α		B3	50.5
V15		[−10−1] γ//[−1−11] α		B2	57.2
V16		[−10−1]γ//[−11−1] α		B1	20.6
V17		[110] γ//[−1−11] α		B3	51.7
V18		[110]γ//[−11−1] α		B2	47.1
V19	(11−1) γ//(011) α	[−110] γ//[−1−11] α	CP4	B3	50.5
V20		[−110] γ//[−11−1] α		B2	57.2
V21		[0−1−1] γ//[−1−11] α		B1	20.6
V22		[0−1−1]]γ//[−11−1] α		B3	47.1
V23		[101] γ//[−1−11] α		B2	57.2
V24		[101]γ//[−11−1] α		B1	21.1

**Table 2:** Chemical composition of experimental material, wt.%.

C	Si	Mn	P	S	Ni	Cr	Cu	Fe
0.300	0.793	0.951	0.011	0.006	0.100	0.090	0.088	Bal.

**Table 3:** Statistical analysis of grain boundary proportions and densities for different steels. F, fraction; D, density.

Steels	F_LAGBs_	F_PAGBs_	F_HAGBs_	D_LAGBs_	D_PAGBs_	D_HAGBs_
RH steel	27%	11%	62%	0.819 μm^−1^	0.345 μm^−1^	1.941 μm^−1^
CH steel	28%	5%	67%	0.754 μm^−1^	0.128 μm^−1^	1.806 μm^−1^

## Data Availability

The original contributions presented in the study are included in the article; further inquiries can be directed to the corresponding author.

## References

[1] Junkui L., Zhinan Y., Hua M., Chen C., Fucheng Z. (2023). A Medium-C Martensite Steel with 2.6 GPa Tensile Strength and Large Ductility. Scr. Mater..

[2] Li Y., Yuan G., Li L., Kang J., Yan F., Du P., Raabe D., Wang G. (2023). Ductile 2-GPa Steels with Hierarchical Substructure. Science.

[3] Liu L., Yu Q., Wang Z., Ell J., Huang M.X., Ritchie R.O. (2020). Making Ultrastrong Steel Tough by Grain-Boundary Delamination. Science.

[4] Wang Z., Huang M.X. (2020). Optimising the Strength-Ductility-Toughness Combination in Ultra-High Strength Quenching and Partitioning Steels by Tailoring Martensite Matrix and Retained Austenite. Int. J. Plast..

[5] He B.B., Hu B., Yen H.W., Cheng G.J., Wang Z.K., Luo H.W., Huang M.X. (2017). High Dislocation Density–Induced Large Ductility in Deformed and Partitioned Steels. Science.

[6] Morito S., Huang X., Furuhara T., Maki T., Hansen N. (2006). The Morphology and Crystallography of Lath Martensite in Alloy Steels. Acta Mater..

[7] Zou Y., Ding H., Zhang Y., Tang Z. (2022). Microstructural Evolution and Strain Hardening Behavior of a Novel Two-Stage Warm Rolled Ultra-High Strength Medium Mn Steel with Heterogeneous Structures. Int. J. Plast..

[8] Morsdorf L., Emelina E., Gault B., Herbig M., Tasan C.C. (2021). Carbon Redistribution in Quenched and Tempered Lath Martensite. Acta Mater..

[9] Macchi J., Teixeira J., Danoix F., Geandier G., Denis S., Bonnet F., Allain S.Y.P. (2024). Impact of Carbon Segregation on Transition Carbides and Cementite Precipitation during Tempering of Low Carbon Steels: Experiments and Modeling. Acta Mater..

[10] Xing X., Huang S., Li L., Ouyang J., Gao J., Chen S., Peng Z. (2023). Optimizing Dislocation Strengthening in High-Strength Medium-Carbon Steel via Fast Induction Heating Quenching & Tempering. J. Mater. Res. Technol..

[11] Komotori J., Shimizu M., Misaka Y., Kawasaki K. (2001). Fatigue Strength and Fracture Mechanism of Steel Modified by Super-Rapid Induction Heating and Quenching. Int. J. Fatigue.

[12] Matlock D.K., Kang S., De Moor E., Speer J.G. (2020). Applications of Rapid Thermal Processing to Advanced High Strength Sheet Steel Developments. Mater. Charact..

[13] Denand B., Esin V.A., Dehmas M., Geandier G., Denis S., Sourmail T., Aeby-Gautier E. (2020). Carbon Content Evolution in Austenite during Austenitization Studied by in Situ Synchrotron X-Ray Diffraction of a Hypoeutectoid Steel. Materialia.

[14] Jacot A., Rappaz M. (1997). A Two-Dimensional Diffusion Model for the Prediction of Phase Transformations: Application to Austenitization and Homogenization of Hypoeutectoid Fe-C Steels. Acta Mater..

[15] Xu Z., Shen X., Allam T., Song W., Bleck W. (2022). Austenite Transformation and Deformation Behavior of a Cold-Rolled Medium-Mn Steel under Different Annealing Temperatures. Mater. Sci. Eng. A.

[16] Liang C., Song G., Wang W., Sohn I., Zeng J. (2024). In Situ Observation of the Austenite to Ferrite Transformation in Low-Carbon Steels from Different Initial Phases at Defined Cooling Rates. J. Mater. Res. Technol..

[17] Yuan Q., Ren J., Mo J., Zhang Z., Tang E., Xu G., Xue Z. (2023). Effects of Rapid Heating on the Phase Transformation and Grain Refinement of a Low-Carbon Mciroalloyed Steel. J. Mater. Res. Technol..

[18] Tan X., Lu W., Rao X. (2022). Effect of Ultra-Fast Heating on Microstructure and Mechanical Properties of Cold-Rolled Low-Carbon Low-Alloy Q&P Steels with Different Austenitizing Temperature. Mater. Charact..

[19] Nishiyama Z. (1978). Introduction to Martensite and Martensitic Transformation. Martensitic Transformation.

[20] Stormvinter A., Miyamoto G., Furuhara T., Hedström P., Borgenstam A. (2012). Effect of Carbon Content on Variant Pairing of Martensite in Fe–C Alloys. Acta Mater..

[21] Kitahara H., Ueji R., Tsuji N., Minamino Y. (2006). Crystallographic Features of Lath Martensite in Low-Carbon Steel. Acta Mater..

[22] Morito S., Tanaka H., Konishi R., Furuhara T., Maki T. (2003). The Morphology and Crystallography of Lath Martensite in Fe-C Alloys. Acta Mater..

[23] Kaneshita T., Miyamoto G., Furuhara T. (2017). Variant Selection in Grain Boundary Nucleation of Bainite in Fe-2Mn-C Alloys. Acta Mater..

[24] Gao X., Wang H., Li J., Lv M., Wu Z., Li Y., Sha G., Ren H. (2022). Cerium-Alloyed Ultra-High Strength Maraging Steel with Good Ductility: Experiments, First-Principles Calculations and Phase-Field Simulations. Mater. Sci. Eng. A..

[25] Zhang X., Miyamoto G., Toji Y., Nambu S., Koseki T., Furuhara T. (2018). Orientation of Austenite Reverted from Martensite in Fe-2Mn-1.5Si-0.3C Alloy. Acta Mater..

[26] Takayama N., Miyamoto G., Furuhara T. (2012). Effects of Transformation Temperature on Variant Pairing of Bainitic Ferrite in Low Carbon Steel. Acta Mater..

[27] Wu B.B., Wang X.L., Wang Z.Q., Zhao J.X., Jin Y.H., Wang C.S., Shang C.J., Misra R.D.K. (2019). New Insights from Crystallography into the Effect of Refining Prior Austenite Grain Size on Transformation Phenomenon and Consequent Mechanical Properties of Ultra-High Strength Low Alloy Steel. Mater. Sci. Eng. A..

[28] Zhao J., Hou Z., Wang B., Yang H., Wang Y., Chang Z., Zhang L., Wu G., Huang X. (2024). Effect of Austenitizing Temperature on Variant Selection of Martensite Transformation in a 2 GPa Grade Steel. J. Mater. Res. Technol..

[29] Yin T.W., Shen Y.F., Jia N., Li Y.J., Xue W.Y. (2023). Controllable Selection of Martensitic Variant Enables Concurrent Enhancement of Strength and Ductility in a Low-Carbon Steel. Int. J. Plast..

[30] Sun M.Y., Wang X.L., Wang Z.Q., Wang X.M., Li X.C., Yan L., Misra R.D.K. (2020). The Critical Impact of Intercritical Deformation on Variant Pairing of Bainite/Martensite in Dual-Phase Steels. Mater. Sci. Eng. A.

[31] Wu B.B., Wang Z.Q., Wang X.L., Xu W.S., Shang C.J., Misra R.D.K. (2019). Toughening of Martensite Matrix in High Strength Low Alloy Steel: Regulation of Variant Pairs. Mater. Sci. Eng. A.

[32] Niessen F., Nyyssönen T., Gazder A.A., Hielscher R. (2022). Parent Grain Reconstruction from Partially or Fully Transformed Microstructures in MTEX. J. Appl. Crystallogr..

[33] Azizi-Alizamini H., Militzer M., Poole W.J. (2011). Austenite Formation in Plain Low-Carbon Steels. Metall. Mater. Trans. A.

[34] Feng H., Wang H., Li H., Zhu H., Zhang S., Jiang Z. (2025). Parent Austenite Grain Reconstruction in Martensitic Steel. J. Mater. Sci. Technol..

[35] Aydogan E., Anderoglu O., Maloy S.A., Livescu V., Gray G.T., Perez-Bergquist S., Williams D.J. (2016). Effect of Shock Loading on the Microstructure, Mechanical Properties and Grain Boundary Characteristics of HT-9 Ferritic/Martensitic Steels. Mater. Sci. Eng. A.

[36] Mao C., Liu C., Yu L., Li H., Liu Y. (2021). Discontinuous Lath Martensite Transformation and Its Relationship with Annealing Twin of Parent Austenite and Cooling Rate in Low Carbon RAFM Steel. Mater. Des..

[37] Sobti A., Mondal K., Kumar Ray R., Sankaran S. (2024). Influencing TRIP Threshold and Variant Pairing through Minor Cold and Cryo-Rolling in Bainitic Steel. Materialia.

[38] Morito S., Pham A.H., Hayashi T., Ohba T. (2015). Block Boundary Analyses to Identify Martensite and Bainite. Mater. Today: Proc..

[39] Archie F., Zaefferer S. (2018). On Variant Selection at the Prior Austenite Grain Boundaries in Lath Martensite and Relevant Micro-Mechanical Implications. Mater. Sci. Eng. A.

[40] Cayron C., Barcelo F., de Carlan Y. (2010). The Mechanisms of the Fcc–Bcc Martensitic Transformation Revealed by Pole Figures. Acta Mater..

[41] Nambu S., Shibuta N., Ojima M., Inoue J., Koseki T., Bhadeshia H.K.D.H. (2013). In Situ Observations and Crystallographic Analysis of Martensitic Transformation in Steel. Acta Mater..

[42] Morsdorf L., Tasan C.C., Ponge D., Raabe D. (2015). 3D Structural and Atomic-Scale Analysis of Lath Martensite: Effect of the Transformation Sequence. Acta Mater..

[43] Miyamoto G., Iwata N., Takayama N., Furuhara T. (2012). Quantitative Analysis of Variant Selection in Ausformed Lath Martensite. Acta Mater..

[44] Wu B., Zhou T., Wang Z., Wang X., Shang C., Yu Y., Elhefnawey M., Li L. (2024). Effects of Carbon Concentration and Transformation Temperature on Incomplete Transformation and Crystallography of Low-Carbon Bainitic Steel. J. Mater. Res. Technol..

[45] Ji H., Wang J., Wang Z., Li Y. (2024). Effect of Martensitic Variant Selection on the Crystallographic Features of Bainite/Martensite Multiphase Structure of 42CrMo Steel. J. Mater. Res. Technol..

